# Recent advances in understanding and diagnosing hepatitis B virus infection

**DOI:** 10.12688/f1000research.8983.1

**Published:** 2016-09-06

**Authors:** Slim Fourati, Jean-Michel Pawlotsky

**Affiliations:** 1 National Reference Center for Viral Hepatitis B, C, and D, Department of Virology, Hôpital Henri Mondor, Université Paris-Est, Créteil, France; 2 INSERM U955, Créteil, France

**Keywords:** hepatitis B virus, receptor, antiviral drugs, HBV DNA, HBs antigen

## Abstract

Hepatitis B virus (HBV) infects approximately 240 million individuals worldwide. Recent advances in the virology, immunopathogenesis, and diagnosis of HBV infection are summarized in this review article. The identification of a hepatocyte-specific cellular receptor for HBV, the sodium taurocholate co-transporting polypeptide (NTCP), made it possible to develop reliable cell culture systems and better understand the early steps of the viral lifecycle. Viral and host factors involved in covalently closed circular DNA synthesis, stability, and transcriptional regulation have also been identified and provide potential targets for new drugs. Based on recent evidence showing trained immunity in immune-tolerant patients, the immune tolerance and immune clearance phases have been renamed the non-inflammatory and inflammatory phases, respectively. New diagnostic and monitoring tools are now available, including rapid diagnostic tests for hepatitis B surface antigen (HBsAg) detection, HBsAg quantification assays, anti-HBc antibody quantification assays, an HBV core-related antigen (HBcrAg) quantification test, new HBV DNA detection and quantification assays, and an HBV RNA quantification test. Their clinical utility is under study. Finally, new antiviral and immune modulation approaches are in the preclinical or early clinical developmental stages, with the goal to achieve functional cure or ideally (if possible) eradication of HBV infection.

## Introduction

Hepatitis B virus (HBV) is responsible for the most frequent chronic liver disease of infectious origin in man, with approximately 240 million chronically infected individuals worldwide. Chronic hepatitis B results in more than 600,000 deaths annually from the complications of end-stage liver disease and hepatocellular carcinoma (HCC) ^1^. The vast majority of new HBV infections occurs in highly endemic regions, such as China, Southeast Asia, and sub-Saharan Africa. HBV transmission can be mother-to-infant, person-to-person in young children (through open cuts and scratches) and adults, sexual, nosocomial, or blood-borne (for instance through sharing of infected needles or drug preparation materials), depending on the prevalence and risk groups in the area.

In the last decade, several factors have changed the worldwide dynamics of HBV epidemiology, including massive population migrations from highly endemic areas and the implementation of preventive strategies, screening policies, and public education. Mostly due to the implementation of prophylactic vaccination and global improvement in the socioeconomic conditions in highly endemic areas, a slow decline of hepatitis B surface antigen (HBsAg) carriage prevalence has been observed, despite the fact that the absolute number of HBV carriers is increasing owing to the increasing world population (estimated at 233 million in 1990 versus 248 million in 2010) ^2^.

The natural history of HBV infection results from a complex dynamic interaction between the virus and its host’s immune defenses ^3^. During acute HBV infection in adults, a broad, vigorous immune response results in viral clearance associated with acute, self-limited inflammatory liver disease in most cases ^4^. In contrast, patients who become chronic HBV carriers, which most commonly occurs from perinatal or early childhood infection, fail to mount efficient innate and adaptive immune responses against HBV ^5^.

Recent years have witnessed significant progress in our understanding of the virologic and immunopathologic aspects of HBV infection, leading the field to reconsider principles that had been established decades ago. Here, we review the most relevant advances in our understanding of HBV pathogenesis and novel virologic tools useful to screen and diagnose HBV-infected patients and optimize their management.

## Recent advances in HBV virology and immunopathology

### New findings in HBV virology

HBV belongs to the *Hepadnaviridae* family. The virus has a circular, partly double-stranded DNA genome. The HBV lifecycle is complex. It starts with attachment of the virus to heparan sulfate proteoglycans, followed by virus binding to a recently identified hepatocyte-specific cellular receptor, the sodium taurocholate co-transporting polypeptide (NTCP) ^6^. The identification of NTCP, a key bile acid transporter expressed by liver cells, as a critical mediator of cellular entry of HBV and hepatitis delta virus (HDV), a viroid using empty HBV envelopes for its infection, paves the way for the development of reliable cell culture systems and a better understanding of the early steps of HBV and HDV infection ^6– 8^.

The pre-S1 region of the HBV envelope proteins appears to bind the extracellular loops of NTCP, triggering endocytosis of the receptor-virus complex prior to transfer of the HBV nucleocapsid (or the HDV ribonucleoprotein complex) to the nucleus ^9^. Early steps of the HBV lifecycle, including HBV membrane fusion, uncoating, and translocation of HBV relaxed circular DNA (rcDNA) to the nucleus, remain poorly understood. In the nucleus, rcDNA is converted into covalently closed circular DNA (cccDNA), the template for the transcription of all viral mRNAs and pregenomic RNA (pgRNA). The transcriptional activity of cccDNA is regulated by epigenetic modifications (e.g., histone acetylations and methylations) and by the HBx protein ^10^. Viral and host factors involved in cccDNA synthesis, stability, and transcriptional regulation have been identified and provide potential targets for drugs aimed at functionally curing HBV infection. For instance, the discovery that tyrosyl-DNA phosphodiesterase 2 is implicated in the first step of cccDNA formation makes it an interesting target for future eradication strategies ^11^. Alternatively, rendering cccDNA transcriptionally inactive, i.e. “locking” HBV cccDNA by means of hyperchromatination, has been suggested as a means to achieve functional cure ^12^.

Virologic factors, such as the HBV genotype, can influence the course of chronic hepatitis B. Genotypes A and D are mainly found in North America, Europe, and Northern and Eastern Africa, while genotypes B and C are dominant in Asia. Individuals infected with genotypes A1, C, B2–5, and F1 showed accelerated progression to cirrhosis and HCC ^13^, whereas genotypes A and B have been associated with a better response to interferon (IFN) alpha therapy than genotypes C and D ^14^.

### New findings in HBV immunology

HBV-infected patients who fail to mount a vigorous and coordinated innate and adaptive immune response develop chronic HBV carriage and are at risk of developing chronic hepatitis B and its complications. The risk of chronicity is related to the patient’s age at infection. Indeed, progression to chronic infection occurs in approximately 90–95% of patients infected perinatally, approximately 30% of patients infected under the age of 5 years, and rarely in patients infected as adults ^15^.

The natural history of chronic HBV infection is not yet fully understood. It results from a complex interplay between the virus and the host that evolves over successive, non-obligatory phases of variable duration during the patient’s life ^16^. They classically include the immune tolerance phase, the hepatitis B envelope antigen (HBeAg)-positive immune clearance phase, the inactive (immune control) phase, and the HBeAg-negative immune escape phase. Based on recent evidence showing trained immunity in immune-tolerant patients, the immune tolerance phase and immune clearance phases have been renamed the non-inflammatory and inflammatory phases, respectively ^17^. The different phases can be individualized based on the HBeAg status and the HBV DNA and alanine aminotransferase (ALT) levels and dynamics.

(i) **Non-inflammatory phase (formerly immune tolerant phase):** the non-inflammatory phase is classically observed during the first two or three decades following perinatally acquired infection. It is characterized by the presence of HBeAg, high HBV DNA levels (generally >10 ^7^ log _10_ international units [IU]/mL), ALT levels within the normal range, and no or minimal histologic inflammation and fibrosis. This phase has long been thought to be associated with no adaptive immune response against the virus, HBeAg being considered as a T-cell tolerogen that crosses the placenta and favors perinatal transmission ^18^. However, the concept of immune tolerance has been recently challenged. Indeed, HBV exposure *in utero* induces a state of trained immunity characterized by innate immune cell maturation and type 1 T-helper cell development ^17^. In infected adolescents, early immune responses against HBV antigens are efficacious, although less prone to induce a proinflammatory reaction than in older individuals ^19^. Persistent low-level immune destruction of infected hepatocytes by cytotoxic T lymphocytes during the non-inflammatory phase is possible. It could lead to the selection of adapted clonal hepatocytes resistant to HBV infection, which would explain the progressive decrease of HBV DNA levels over time and the potential triggering of HCC development during this early phase ^20^. In young patients, other components of the immune system, such as immune regulatory factors and natural killer T cells, which are very abundant in the liver, may also play a role. Integration of HBV DNA into the host hepatocyte genome might also occur at a more rapid rate than in the inactive phase, setting the stage for HCC development as infected persons’ age.
(ii) **Inflammatory phase (formerly immune active or immune elimination phase):** the inflammatory phase is characterized by the presence of HBeAg, persistently or intermittently elevated ALT levels, and sometimes fluctuating HBV DNA levels that remain high but lower than in patients at the non-inflammatory phase. The inflammatory phase results from immune-mediated liver damage, characterized by the presence of liver necroinflammation and, often, fibrosis, as assessed by liver biopsy or noninvasive methods. ALT flares are common during this phase. They are generally clinically asymptomatic. Acute hepatitis, sometimes complicated by hepatic decompensation in cirrhotic patients, occurs in approximately 5% of flares, mostly when HBV DNA levels are very high ^21^.
(iii) **Inactive phase:** the inactive phase is characterized by the loss of HBeAg and the appearance of anti-HBe antibodies, ALT levels within the normal range, and HBV DNA levels that fall below 2000 IU/mL, sometimes becoming undetectable ^22^. During the natural course of chronic HBV infection, the timing of HBeAg seroconversion is influenced by a number of factors, including the age at acquisition and the HBV genotype ^23, 24^. The yearly rate of spontaneous HBeAg seroconversion is less than 2% in chronically infected children <3 years old; it increases to reach 12% during adulthood. Patients who seroconvert after the age of 40 years have a higher risk of developing cirrhosis and HCC, as compared to patients with earlier seroconversion ^25^. Spontaneous reversion to HBeAg positivity is possible in up to 8% of patients ^26^. HBeAg seroconversion can also be induced by antiviral therapy, when blocking of viral replication allows the immune system to take control of infection.
(iv) **Immune escape phase (HBeAg-negative inflammatory phase):** the immune escape phase is observed in HBeAg-negative patients who cannot express HBeAg owing to genome mutations in the basal core promoter and/or in the precore region of the pre-C/C gene. These patients have active liver disease, with elevated fluctuating ALT levels, and persistent viral replication, with HBV DNA levels classically lower than in HBeAg-positive patients at the inflammatory phase. Progression to fibrosis and cirrhosis is generally faster in HBeAg-negative than in HBeAg-positive patients with active inflammation. During this phase, there is a direct correlation between HBV DNA levels in serum and the risk of HCC ^26– 28^.

## Recent advances in diagnosis and monitoring of HBV infection

The diagnosis of HBV infection is currently based on the detection of serological markers, including HBsAg, anti-HBs antibodies, anti-HBc antibodies (total or IgM), HBeAg, and anti-HBe antibodies, and on the detection and quantification of HBV DNA in peripheral blood. The diagnosis of acute hepatitis B is based on the concomitant presence of HBsAg and anti-HBc IgM. In chronic HBsAg carriers, the presence or absence of HBeAg, the HBV DNA level, and the ALT level help diagnose the phase of chronic infection (see above).

Recent advances have been made to develop and/or improve serologic and molecular virologic tools useful for global screening, diagnosis, and optimal patient management.

### Rapid diagnostic tests for HBsAg detection

A number of countries, including in resource-limited areas, have implemented large-scale prophylactic vaccination campaigns that are profoundly affecting HBV incidence and related morbidity and mortality. However, the worldwide HBV prevalence remains very high. Because anti-HBV drugs are effective and now affordable in many areas, large-scale screening for infection has become a priority in order to improve access to care for those who are infected and provide vaccination for unprotected individuals.

Screening for HBV infection is based on the detection of HBsAg in peripheral blood. Commercial HBsAg detection based on enzyme-linked immunosorbent assay (ELISA) is often not accessible in resource-limited settings or in marginalized populations. Rapid diagnostic tests (RDTs) have been recently developed. RDTs do not require laboratory infrastructure, are easy to perform with minimal training, and provide conclusive results within a few minutes. These tests can be performed not only with serum or plasma but also with whole-blood collected by fingerstick and, for some of them, with oral fluids. Their sensitivity varies with the test and matrix used, while their specificity is high (close to 100%). Some RDTs were recently shown to have excellent performance for the detection of HBsAg, i.e. VIKIA HBsAg (bioMérieux, Marcy l’Etoile, France), Alere Determine HBsAg (Alere, Inc., Waltham, Massachusetts), or DRW-HBsAg v2 assay (Diagnostics for the Real World, Ltd, Sunnyvale, California) ^29^. Importantly, these tests were shown to be sensitive for the detection of HBsAg mutants missed by some ELISA assays ^29, 30^. Overall, validated HBsAg RDTs are reliable and now represent promising tools for large-scale screening and diagnosis of HBV infection.

### HBsAg quantification

Commercially available techniques, including Elecsys (Roche Diagnostics, Indianapolis, Indiana) and Architect (Abbott Laboratories, Abbott Park, Illinois), are now available to accurately quantify HBsAg. The HBsAg level in serum is believed to reflect the balance between the host immune response and viral replication. The kinetics of HBsAg production are complex and differ at each phase of the natural history of HBV, gradually declining from 4.5 to 2.8 log _10_ IU/mL on average between the non-inflammatory and the inactive phase, respectively ^31^.

HBsAg quantification has several potential indications in clinical practice ^32^. It can be used to better characterize the inactive phase complementary to the HBV DNA level that often fluctuates in patients at the inflammatory phase ^31^. An HBsAg level <1000 IU/mL together with an HBV DNA level <2000 IU/mL were suggested to be associated with the inactive carrier state ^33^. The HBsAg level was also suggested to predict the severity of fibrosis in treatment-naïve HBeAg-positive patients infected with HBV genotypes B and C ^34^. HBeAg-negative patients with low HBV DNA levels but high HBsAg levels were suggested to be at higher risk of HCC ^35^.

There is increasing evidence to suggest that monitoring HBsAg levels prior to and during treatment predicts the sustained response to pegylated IFN alpha-based therapy ^36^. Both an on-treatment decline of HBsAg level >1 log _10_ IU/mL and an HBsAg level <10 IU/mL at week 48 were significantly associated with sustained HBsAg clearance 3 years after pegylated IFN treatment in HBeAg-negative patients ^37^. In another study, reducing the HBsAg level to 1500 IU/mL at week 12 and to 300 IU/mL at week 24 was associated with a high rate of response to pegylated IFN treatment in HBeAg-positive patients ^38^. Finally, in 803 HBeAg-positive patients treated with pegylated IFN, most patients with HBsAg >20,000 IU/mL at week 24 failed to respond to therapy, regardless of the HBV genotype. These authors proposed that treatment should be discontinued in this situation ^39^.

Data are lacking regarding prediction of nucleos(t)ide analogue treatment response by HBsAg levels ^40^. It has been suggested that the addition of pegylated IFN is more likely to lead to an HBsAg loss in patients with chronic hepatitis B who respond to nucleos(t)ide analogue treatment and experience an HBsAg level decrease ^41^. More data are needed to confirm whether this remains true with HBV genotypes found in western areas and to identify time points with good predictive values.

### Anti-HBc antibody quantification

It has been suggested that anti-HBc antibody levels reflect HBV-specific adaptive immunity and could predict the response to antiviral therapies ^42^. Corroborating this hypothesis, the baseline anti-HBc antibody titer was a strong predictor of HBeAg seroconversion in 791 HBeAg-positive patients with chronic hepatitis B receiving either pegylated IFN or nucleos(t)ide analogues ^43^. If confirmed by other studies, this marker could be used for pretreatment stratification.

### HBV core-related antigen (HBcrAg) quantification

Another potentially useful novel marker is the hepatitis B core-related antigen (HBcrAg). HBcrAg detects an amino acid sequence shared by HBeAg and the hepatitis B core protein. Serum HBcrAg levels were reported to correlate with serum HBV DNA and intrahepatic HBV DNA and cccDNA levels as well as with disease activity in early studies ^44^. More recently, HBcrAg levels were strongly associated with the development of HCC ^45^. HBcrAg has also been suggested to be a marker of HBV reinfection after liver transplantation ^46^. Further investigation is needed, especially outside of Asia, where this marker has been exclusively studied thus far.

### New HBV DNA assays

Detection and quantification of HBV DNA levels are essential to diagnose ongoing HBV infection, establish the prognosis of liver disease, estimate the mother-to-child risk of transmission, and guide treatment decisions. HBV DNA quantification is indispensable in monitoring the virologic response to antiviral therapy ^47^. HBV DNA level quantification is currently based on the use of real-time polymerase chain reaction (PCR) and isothermal amplification methods such as transcription-mediated amplification (TMA). Commercially available assays showed excellent analytical sensitivity (lower limit of detection in the order of 10 to 20 IU/mL) and specificity, and a broad dynamic range of linear quantification, fully covering the clinical needs, in a number of studies ^48^.

Next-generation molecular diagnostic tools, such as the real-time TMA-based Aptima HBV Quant assay (Hologic, Bedford, Massachusetts) or the real-time PCR-based VERIS HBV assay (Beckman Coulter, Brea, California), will be available soon. In these assays, all steps, including sample loading, nucleic acid extraction, reaction setup, and amplification, are integrated in a fully streamlined workflow. These assays will enable biologists to deliver results within hours following sampling, allowing for faster clinical decision-making. Random access will guarantee that emergency testing can be performed without disrupting the workflow.

### HBV RNA quantification

HBV RNA can be detected and quantified in infected patients’ blood. In untreated patients, HBV RNA levels strongly correlate with HBV DNA levels ^49, 50^. During nucleos(t)ide analogue therapy, the decrease of serum HBV RNA level was reported to be strongly predictive of subsequent HBeAg seroconversion ^49, 50^. Patients receiving the combination of pegylated IFN and a nucleos(t)ide analogue had a sharper decline of HBV RNA levels than those receiving nucleos(t)ide analogue monotherapy ^49, 50^. If these findings, which suggest that on-treatment HBV RNA level changes have a better predictive value on treatment outcome than changes in HBV DNA or HBsAg levels, are confirmed, HBV RNA monitoring could find an indication in treatment monitoring in clinical practice.

## Recent advances in HBV therapy

Long-term control of HBV replication can now be achieved by finite administration of pegylated IFN when it induces a sustained virologic response, or by lifelong administration of a nucleos(t)ide analogue, such as tenofovir or entecavir. Nucleos(t)ide analogues block HBV replication and the formation of new cccDNAs in the long term without selecting resistant viruses in the vast majority of cases. However, they have no or little effect on the hepatocyte pool of cccDNA, which plays a key role in viral persistence and reactivation if antiviral therapy is interrupted. Therefore, these agents exceptionally yield a functional cure of chronic HBV infection, defined as sustained or durable HBsAg loss associated with anti-HBs antibody seroconversion with undetectable HBV DNA in serum and persistence of transcriptionally inactive cccDNA ^51, 52^. Furthermore, no anti-HBV agent provides definitive eradication, as defined by complete cccDNA elimination.

New antiviral approaches that target various steps and components of the HBV lifecycle, including cccDNA, are currently being investigated, with the hope of achieving functional cure of infection or, if possible, complete viral eradication. These approaches, which have been recently reviewed ^51, 52^, include HBV entry inhibitors, such as Myrcludex B ^TM^, a lipopeptide mimicking the pre-S1 domain that competes with HBV particles for binding to NTCP; cytokines or sequence-specific nucleases that damage or destroy cccDNA; modulators of host cellular epigenetic-modifying enzymes, such as cytokines or inhibitors of viral protein function, that functionally silence cccDNA; cholesterol-conjugated small-interfering RNAs or antisense oligonucleotides blocking viral replication and viral protein expression; RNAse H inhibitors; capsid assembly modulators affecting nucleocapsid assembly, pgRNA encapsidation, and the nuclear functions of HBV core protein (cccDNA regulation and IFN-stimulated gene expression); phosphorothioate oligonucleotides that inhibit HBsAg release; and monoclonal antibodies that decrease circulating HBsAg load ^51, 52^. These agents are at early phases of development, and further preclinical and clinical evaluations will be needed to assess their safety and efficacy.

Therapeutic approaches based on immune modulation are also currently being explored for future use in combination with antiviral options. They have been recently reviewed ^51, 52^ and include innate immune response modulators, such as IFN alpha, TLR7 agonists, or compounds antagonizing cellular inhibitor of apoptosis (cIAP); inducers of HBV-specific T-cell exhaustion, such as PD-1 antagonists, and immunosuppressive cytokines (IL-10 and TGF *β*); engineered redirected T cells and immune effector cells re‐targeted towards HBV‐infected cells; and therapeutic vaccines.

## Conclusion

The control of HBV infection has made considerable progress over the last two decades, with continuous improvement of virologic tools for screening and diagnosis, the implementation of widespread vaccination programs, and the development of effective antiviral therapies that block viral replication in the long term. However, available antiviral strategies do not yield functional cure of chronic HBV infection in most cases, and the risks related to HBV-induced cirrhosis and HCC still represent a major threat for infected patients. Characterization of the early events of HBV-induced tumorigenesis is needed to identify new biomarkers of disease progression and HCC development in order to implement personalized approaches for prevention, screening, and early intervention. New antiviral approaches combining antiviral agents acting on multiple targets in the HBV lifecycle and immune modulation aimed at achieving HBV functional cure or eradication and reducing the burden of HBV-induced liver disease are still under investigation. A concerted action of academic centers and pharmaceutical industries will be warranted to coordinate the development of new treatment strategies that, together with large-scale screening and universal vaccination, will help win the battle against HBV.

## References

[1] GBD 2013 Mortality and Causes of Death Collaborators: Global, regional, and national age-sex specific all-cause and cause-specific mortality for 240 causes of death, 1990-2013: a systematic analysis for the Global Burden of Disease Study 2013. *Lancet.* 2015;385(9963):117–71. 10.1016/S0140-6736(14)61682-2 25530442 PMC4340604

[2] Schweitzer A Horn J Mikolajczyk RT: Estimations of worldwide prevalence of chronic hepatitis B virus infection: a systematic review of data published between 1965 and 2013. *Lancet.* 2015;386(10003):1546–55. 10.1016/S0140-6736(15)61412-X 26231459

[3] Lai CL Ratziu V Yuen MF: Viral hepatitis B. *Lancet.* 2003;362(9401):2089–94. 10.1016/S0140-6736(03)15108-2 14697813

[4] Bertoletti A Tan AT Gehring AJ: HBV-Specific Adaptive Immunity. *Viruses.* 2009;1(2):91–103. 10.3390/v1020091 21994540 PMC3185487

[5] Busca A Kumar A: Innate immune responses in hepatitis B virus (HBV) infection. *Virol J.* 2014;11:22. 10.1186/1743-422X-11-22 24507433 PMC3922976

[6] Yan H Zhong G Xu G: Sodium taurocholate cotransporting polypeptide is a functional receptor for human hepatitis B and D virus. *eLife.* 2012;1:e00049. 10.7554/eLife.00049 23150796 PMC3485615

[7] Allweiss L Dandri M: Experimental *in vitro* and *in vivo* models for the study of human hepatitis B virus infection. *J Hepatol.* 2016;64(1 Suppl):S17–31. 10.1016/j.jhep.2016.02.012 27084033

[8] Ni Y Lempp FA Mehrle S: Hepatitis B and D viruses exploit sodium taurocholate co-transporting polypeptide for species-specific entry into hepatocytes. *Gastroenterology.* 2014;146(4):1070–83. 10.1053/j.gastro.2013.12.024 24361467

[9] Li W Urban S: Entry of hepatitis B and hepatitis D virus into hepatocytes: Basic insights and clinical implications. *J Hepatol.* 2016;64(1 Suppl):S32–40. 10.1016/j.jhep.2016.02.011 27084034 PMC7114860

[10] Belloni L Pollicino T De Nicola F: Nuclear HBx binds the HBV minichromosome and modifies the epigenetic regulation of cccDNA function. *Proc Natl Acad Sci U S A.* 2009;106(47):19975–9. 10.1073/pnas.0908365106 19906987 PMC2775998

[11] Königer C Wingert I Marsmann M: Involvement of the host DNA-repair enzyme TDP2 in formation of the covalently closed circular DNA persistence reservoir of hepatitis B viruses. *Proc Natl Acad Sci U S A.* 2014;111(40):E4244–53. 10.1073/pnas.1409986111 25201958 PMC4209993

[12] Nassal M: HBV cccDNA: viral persistence reservoir and key obstacle for a cure of chronic hepatitis B. *Gut.* 2015;64(12):1972–84. 10.1136/gutjnl-2015-309809 26048673

[13] Kramvis A: Genotypes and genetic variability of hepatitis B virus. *Intervirology.* 2014;57(3–4):141–50. 10.1159/000360947 25034481

[14] Raimondi S Maisonneuve P Bruno S: Is response to antiviral treatment influenced by hepatitis B virus genotype? *J Hepatol.* 2010;52(3):441–9. 10.1016/j.jhep.2009.12.014 20137824

[15] Yim HJ Lok AS: Natural history of chronic hepatitis B virus infection: what we knew in 1981 and what we know in 2005. *Hepatology.* 2006;43(2 Suppl 1):S173–81. 10.1002/hep.20956 16447285

[16] Terrault NA Bzowej NH Chang K: AASLD guidelines for treatment of chronic hepatitis B. *Hepatology.* 2016;63(1):261–83. 10.1002/hep.28156 26566064 PMC5987259

[17] Hong M Sandalova E Low D: Trained immunity in newborn infants of HBV-infected mothers. *Nat Commun.* 2015;6:6588. 10.1038/ncomms7588 25807344 PMC4389241

[18] Milich DR Jones JE Hughes JL: Is a function of the secreted hepatitis B e antigen to induce immunologic tolerance in utero? *Proc Natl Acad Sci U S A.* 1990;87(17):6599–603. 2395863 10.1073/pnas.87.17.6599 PMC54584

[19] Kennedy PT Sandalova E Jo J: Preserved T-cell function in children and young adults with immune-tolerant chronic hepatitis B. *Gastroenterology.* 2012;143(3):637–45. 10.1053/j.gastro.2012.06.009 22710188

[20] Zoulim F Mason WS: Reasons to consider earlier treatment of chronic HBV infections. *Gut.* 2012;61(3):333–6. 10.1136/gutjnl-2011-300937 22147510

[21] Jeng WJ Sheen IS Liaw YF: Hepatitis B virus DNA level predicts hepatic decompensation in patients with acute exacerbation of chronic hepatitis B. *Clin Gastroenterol Hepatol.* 2010;8(6):541–5. 10.1016/j.cgh.2010.02.023 20298811

[22] Papatheodoridis GV Manolakopoulos S Liaw YF: Follow-up and indications for liver biopsy in HBeAg-negative chronic hepatitis B virus infection with persistently normal ALT: a systematic review. *J Hepatol.* 2012;57(1):196–202. 10.1016/j.jhep.2011.11.030 22450396

[23] Chu CJ Hussain M Lok AS: Hepatitis B virus genotype B is associated with earlier HBeAg seroconversion compared with hepatitis B virus genotype C. *Gastroenterology.* 2002;122(7):1756–62. 10.1053/gast.2002.33588 12055581

[24] Liu CJ Kao JH: Global perspective on the natural history of chronic hepatitis B: role of hepatitis B virus genotypes A to J. *Semin Liver Dis.* 2013;33(2):97–102. 10.1055/s-0033-1345716 23749665

[25] Chen YC Chu CM Liaw YF: Age-specific prognosis following spontaneous hepatitis B e antigen seroconversion in chronic hepatitis B. *Hepatology.* 2010;51(2):435–44. 10.1002/hep.23348 19918971

[26] Lok AS Lai CL Wu PC: Spontaneous hepatitis B e antigen to antibody seroconversion and reversion in Chinese patients with chronic hepatitis B virus infection. *Gastroenterology.* 1987;92(6):1839–43. 10.1016/0016-5085(87)90613-5 3569757

[27] Chen CF Lee WC Yang HI: Changes in serum levels of HBV DNA and alanine aminotransferase determine risk for hepatocellular carcinoma. *Gastroenterology.* 2011;141(4):1240–8, 1248.e1-2. 10.1053/j.gastro.2011.06.036 21703214

[28] Chen CJ Yang HI Su J: Risk of hepatocellular carcinoma across a biological gradient of serum hepatitis B virus DNA level. *JAMA.* 2006;295(1):65–73. 10.1001/jama.295.1.65 16391218

[29] Chevaliez S Challine D Naija H: Performance of a new rapid test for the detection of hepatitis B surface antigen in various patient populations. *J Clin Virol.* 2014;59(2):89–93. 10.1016/j.jcv.2013.11.010 24355522

[30] Servant-Delmas A Ly TD Hamon C: Comparative Performance of Three Rapid HBsAg Assays for Detection of HBs Diagnostic Escape Mutants in Clinical Samples. *J Clin Microbiol.* 2015;53(12):3954–5. 10.1128/JCM.02117-15 26378274 PMC4652127

[31] Brunetto MR Oliveri F Colombatto P: Hepatitis B surface antigen serum levels help to distinguish active from inactive hepatitis B virus genotype D carriers. *Gastroenterology.* 2010;139(2):483–90. 10.1053/j.gastro.2010.04.052 20451520

[32] Chevaliez S: Is HBsAg quantification ready, for prime time? *Clin Res Hepatol Gastroenterol.* 2013;37(6):559–63. 10.1016/j.clinre.2013.07.004 23932705

[33] Martinot-Peignoux M Lapalus M Asselah T: The role of HBsAg quantification for monitoring natural history and treatment outcome. *Liver Int.* 2013;33(Suppl 1):125–32. 10.1111/liv.12075 23286856

[34] Martinot-Peignoux M Carvalho-Filho R Lapalus M: Hepatitis B surface antigen serum level is associated with fibrosis severity in treatment-naïve, e antigen-positive patients. *J Hepatol.* 2013;58(6):1089–95. 10.1016/j.jhep.2013.01.028 23369792

[35] Tseng TC Liu CJ Yang HC: High levels of hepatitis B surface antigen increase risk of hepatocellular carcinoma in patients with low HBV load. *Gastroenterology.* 2012;142(5):1140–1149.e3; quiz e13–4. 10.1053/j.gastro.2012.02.007 22333950

[36] Ma H Yang RF Wei L: Quantitative serum HBsAg and HBeAg are strong predictors of sustained HBeAg seroconversion to pegylated interferon alfa-2b in HBeAg-positive patients. *J Gastroenterol Hepatol.* 2010;25(9):1498–506. 10.1111/j.1440-1746.2010.06282.x 20796146

[37] Brunetto MR Moriconi F Bonino F: Hepatitis B virus surface antigen levels: a guide to sustained response to peginterferon alfa-2a in HBeAg-negative chronic hepatitis B. *Hepatology.* 2009;49(4):1141–50. 10.1002/hep.22760 19338056

[38] Chan HL: Response-guided therapy by hepatitis B surface antigen level for peginterferon therapy: what is next? *J Gastroenterol Hepatol.* 2012;27(3):420–1. 10.1111/j.1440-1746.2012.07062.x 22353347

[39] Sonneveld MJ Hansen BE Piratvisuth T: Response-guided peginterferon therapy in hepatitis B e antigen-positive chronic hepatitis B using serum hepatitis B surface antigen levels. *Hepatology.* 2013;58(3):872–80. 10.1002/hep.26436 23553752

[40] Chevaliez S Hezode C Bahrami S: Long-term hepatitis B surface antigen (HBsAg) kinetics during nucleoside/nucleotide analogue therapy: finite treatment duration unlikely. *J Hepatol.* 2013;58(4):676–83. 10.1016/j.jhep.2012.11.039 23219442

[41] Wu Z Sheng J Li L: Improvement of HBsAg loss by additional PEG-IFN in nucleosides analogs treated chronic hepatitis B patients. *Hepatology.* 2012;56(Suppl 1):404A.

[42] Yuan Q Song LW Liu CJ: Quantitative hepatitis B core antibody level may help predict treatment response in chronic hepatitis B patients. *Gut.* 2013;62(1):182–4. 10.1136/gutjnl-2012-302656 22698651

[43] Fan R Sun J Yuan Q: Baseline quantitative hepatitis B core antibody titre alone strongly predicts HBeAg seroconversion across chronic hepatitis B patients treated with peginterferon or nucleos(t)ide analogues. *Gut.* 2016;65(2):313–20. 10.1136/gutjnl-2014-308546 25586058 PMC4752655

[44] Wong DK Tanaka Y Lai CL: Hepatitis B virus core-related antigens as markers for monitoring chronic hepatitis B infection. *J Clin Microbiol.* 2007;45(12):3942–7. 10.1128/JCM.00366-07 17942661 PMC2168562

[45] Tada T Kumada T Toyoda H: HBcrAg predicts hepatocellular carcinoma development: An analysis using time-dependent receiver operating characteristics. *J Hepatol.* 2016;65(1):48–56. 10.1016/j.jhep.2016.03.013 27034253

[46] Matsuzaki T Tatsuki I Otani M: Significance of hepatitis B virus core-related antigen and covalently closed circular DNA levels as markers of hepatitis B virus re-infection after liver transplantation. *J Gastroenterol Hepatol.* 2013;28(7):1217–22. 10.1111/jgh.12182 23432697

[47] European Association For The Study Of The Liver: EASL clinical practice guidelines: Management of chronic hepatitis B virus infection. *J Hepatol.* 2012;57(1):167–85. 10.1016/j.jhep.2012.02.010 22436845

[48] Pawlotsky J Dusheiko G Hatzakis A: Virologic monitoring of hepatitis B virus therapy in clinical trials and practice: recommendations for a standardized approach. *Gastroenterology.* 2008;134(2):405–15. 10.1053/j.gastro.2007.11.036 18242209 PMC2676233

[49] Jansen L Kootstra NA van Dort KA: Hepatitis B Virus Pregenomic RNA Is Present in Virions in Plasma and Is Associated With a Response to Pegylated Interferon Alfa-2a and Nucleos(t)ide Analogues. *J Infect Dis.* 2016;213(2):224–32. 10.1093/infdis/jiv397 26216905

[50] van Bömmel F Bartens A Mysickova A: Serum hepatitis B virus RNA levels as an early predictor of hepatitis B envelope antigen seroconversion during treatment with polymerase inhibitors. *Hepatology.* 2015;61(1):66–76. 10.1002/hep.27381 25132147

[51] Revill P Testoni B Locarnini S: Global strategies are required to cure and eliminate HBV infection. *Nat Rev Gastroenterol Hepatol.* 2016;13(4):239–48. 10.1038/nrgastro.2016.7 26907881

[52] Zeisel MB Lucifora J Mason WS: Towards an HBV cure: state-of-the-art and unresolved questions--report of the ANRS workshop on HBV cure. *Gut.* 2015;64(8):1314–26. 10.1136/gutjnl-2014-308943 25670809

